# Prodigiosin inhibits cholangiocarcinoma cell proliferation and induces apoptosis via suppressing SNAREs-dependent autophagy

**DOI:** 10.1186/s12935-021-02355-3

**Published:** 2021-12-09

**Authors:** Dijie Zheng, Shiyu Chen, Kun Cai, Linhan Lei, Chunchen Wu, Chengyi Sun, Yazhu Deng, Chao Yu

**Affiliations:** 1 Guizhou Medical University, Guiyang, 550004 China; 2 School of Clinical Medicine, Guizhou Medical University, Guiyang, China; 3 School of Basic Medical Sciences, Guizhou Medical University, Guiyang, China; 4 Department of Hepatobiliary Surgery, The Affiliated Hospital of Guizhou Medical University, Guiyang, China; 5 Guizhou Provincial Institute of Hepatobiliary, Pancreatic and Splenic Diseases, Guiyang, China; 6 Key Laboratory of Liver, Gallbladder, Pancreas and Spleen of Guizhou Medical University, Guiyang, China; 7 Department of General Surgery, The Affiliated Hospital of Guizhou Medical University, Guiyang, China

**Keywords:** Prodigiosin, SNAREs complex, Cholangiocarcinoma, Autophagy, Proliferation

## Abstract

**Background:**

Prodigiosin (PG), a natural red pigment produced by numerous bacterial species, has been a eye-catching research point in recent years for its anticancer activity. However, the role of PG in the cancer biology of cholangiocarcinoma (CCA) remains vague.

**Methods:**

The proliferation of CCA cells was detected by Cell Counting Kit-8(CCK-8), Colony formation assay and 5-ethynyl-2′-deoxyuridine (EdU) assay. Cell apoptosis was evaluated by flow cytometry assay and western blot assay. The effects of PG or SNAREs on cell autophagy were measured by autophagy flux assay and western blot assay. Xenograft mouse models were used to assess the role of PG in CCA cells in vivo.

**Results:**

PG could inhibit the proliferation and viability of CCA cells in a concentration- and time-dependent manner via suppressing the late stage of autophagy. Mechanistically, PG inhibits the fusion of autophagosomes and lysosomes by blocking STX17 and SNAP29, components of soluble N-ethyl-maleimide-sensitive factor attachment protein receptors (SNAREs)complex. When STX17 and SNAP29 were overexpressed, the inhibitory effect of PG on CCA cells autophagy was relieved. In addition, PG showed obvious inhibitory effects on cancer cell viability but no toxic effects on organs in xenotransplantation models.

**Conclusion:**

Taken together, our results demonstrated that PG inhibits CCA cell proliferation via suppressing SNAREs-dependent autophagy, implying that PG could be a potential chemotherapy drug for advanced CCA.

## Introduction

Cholangiocarcinoma (CCA)is a malignancy originating from biliary epithelial cells. The morbidity of CCA is high and ranks second in all liver malignancies [1]. In the past thirty years, the incidence of CCA worldwide has been increasing steadily [2]. Surgery is the most effective treatment for early CCA [3]. Since patients with CCA usually would not exhibit obvious symptoms in early stage, while the patients could be already in advanced stage when firstly diagnosed. Meanwhile, epidemiological studies reported the 5-year survival rate of patients with CCA is < 10% [4, 5]. In addition, there is a lack of effective methods for the treatment of patients without surgery. Thus, it is of great value to develop novel anti-tumor agents for patients with late stage CCA.

The commonly used chemotherapy regimen for CCA is cisplatin combined with gemcitabine, while poor tolerance for side effects and poor treatment efficacy makes it far beyond satisfactory [6]; Recent studies have shown that natural products have certain applicable value in the prevention and treatment of cancer [7]. Prodigiosin (PG) is a general term for a class of natural red pigments, which is a biologically active secondary metabolite produced by actinomycetes, *Serratia marcescens*, or *Pseudomona* [8]. In addition, PG was shown to exhibit favorable biological activities in anti-bacterial [9], and anti-tumor activities, which has been proven to be an effective apoptotic agent for colon cancer, and lung cancer [10, 11]. Although there are numerous evidences regarding the anti-tumor activity of PG, studies on the underlying mechanism are scarce.

Accumulated researches demonstrated that natural products usually play anti-tumor activity by regulating various cell death manners, including apoptosis, autophagy, ferroptosis and so on [12–14]. Meanwhile, targeting autophagy process was known as an effective strategy to assist other drugs therapeutic effects. Autophagy is a key intracellular degradation process and is essential for degradation of long-lived proteins, misfolded proteins, and damaged organelles to maintain cell metabolism [15]. Dysregulation of autophagy has been reported to be associated with diverse pathologies such as cancer, inflammation [16, 17]. The complete autophagy consists of four steps: the formation of phagocytic vesicles, the formation of autophagosomes, the fusion of autophagosomes and lysosomes to form autolysosomes, and the degradation of autolysosomes [18]. The fusion of autophagosomes and lysosomes was considered the vital process in the late stage of autophagy, which was regulated by SNAREs complex [19]. Research has shown that SNAREs complex are transmembrane proteins, which are composed of STX17 (syntaxin 17), SNAP29 (synaptosome associated protein 29 kDa), and VAMP8 (vesicle-associated membrane protein 8) [20]. Previous studies indicate that the SNAREs positively regulates autophagic processes [21]. In addition, inhibition of SNAREs complex is associated with different biological effects, which includes activation of autophagic cell death and induction of cytoprotective autophagy [22, 23]. However, the mechanism behind SNAREs complex associated PG-regulated autophagy remains unknown. Further studies are needed to explore the effects of SNAREs complex on autophagy, especially its role in regulating cell death and cytoprotection.

In this work, we demonstrated the anti-cancer activity of PG in vitro and in vivo. We also find that inhibiting autophagy leads to the suppression of proliferation capacity in CCA cells. Finally, the underlying mechanisms of autophagy inhibited by PG in CCA cells was demonstrated to be correlated with the SNAREs complex.

## Materials and methods

### Cell culture and reagent

Human CCA cancer cell lines (TFK-1, HuCCT-1, HUH28, QBC939, RBE, and CCLP-1) were purchased from the American Type Culture Collection (ATCC; Manassas, VA, USA) and cultured in RPMI1640 medium supplemented with 10% fetal bovine serum (FBS, Biological Industries, Israel) at 37 °C. STX17 overexpression (STX17), SNAP29 overexpression (SNAP29) and negative-control (Vector) lentiviruses were purchased from Genechem (Shanghai, China). All transfections were performed base on the instructions of manufacturer. All cell lines have been tested for mycoplasma contamination before conducting this study. Prodigiosin was purchased from Sigma-aldrich (CAS: 56144-17-3, USA) and stored at –20 °C.

### Colony formation assay

To analyze colony formation, HuCCT-1 and TFK-1 cells were seeded at a density of 500 cells/well in six-well plates. After 24 h, the cells were exposed to different concentrations (0, 150, 300, 600, and 1200 nM) of PG. Then the cultured cells were incubated in a humidified atmosphere of 5% CO_2_ at 37 °C for 14 days. Subsequently, the plates were washed with phosphate-buffered saline (PBS), fixed with methanol for 15 min, and stained with 0.25% crystal violet for 20 min.

### Cell viability assay

For cell viability assay, 1000 cells/well were plated into 96-well plates. Cells were exposed to different concentrations of PG (0, 150, 300, 600, and 1200 nM), and then incubated for 24 h. Subsequently, 10 µL of CCK-8 reagent were added to each well. Then the plates were incubated for 3 h in an atmosphere of 5% CO_2_ and 37℃. The optical density was measured at 450 nm on a microplate reader (Molecular Devices, Sunnyvale, CA, USA).

### EdU assay

CCA cells were seeded on a 24-well plate at 8000 cells/well and cultivated. When the cell confluency reached 60%, EdU kit (Ruibo Biological Co., Ltd, Guangzhou, China) was applied for staining. The proliferation rate was calculated under a fluorescence microscope.

### Flow cytometric analysis of apoptosis

HuCCT-1 and TFK-1 cells were plated in 24-well plates (6 × 10^4^ cells/well) and then treated with PG for 24 h. Cells were then collected for subsequent detection using an Annexin VPE/7-AAD Apoptosis Detection Kit (BD Biosciences, San Diego, CA, USA) for apoptosis analysis. FACSCalibur system (BD, Franklin Lakes, NJ, USA) was used for cell analysis. Data analysis was performed using FlowJo software (BD Biosciences).

### Transmission electron microscopy (TEM)

HuCCT-1 and TFK-1 cells were treated with PG (600 nM for 24 h). Then 4% glutaraldehyde and 1% osmium tetroxide were used to fix the cells. a graded series of ethanol for dehydration and propylene oxide for infiltration. Samples were then embedded. Ultramicrotome (Leica) was used to cut the embedded samples into 50 nm thick sections. Finally the slices were double-stained with 3% uranyl acetate and lead citrate. We used TEM (Hitachi HT7700, Tokyo, Japan) for sample observation.

### Autophagy flux analysis

HuCCT-1 and TFK-1 cells transfection was performed using mRFP-GFP-LC3 adenoviral vectors purchased from HanBio Technology (Shanghai, China). Cells incubation was performed under PG (600 nM) treatment at 37 °C for 24 h. When autophagosomes and lysosomes fused, the pH in the autophagosome was altered, and then GFP was disrupted and quenched. The autophagy flux was observed using a Zeiss LSM710 confocal microscope (Jena, Germany, Carl Zeiss).

### Western blot

Cells were harvested and lysed in radioimmunoprecipitation (RIPA) lysis buffer. An equivalent of 20 mg of proteins was separated on 10% Sodium dodecyl sulfate- polyacrylamide gel electrophoresis (SDS-PAGE) and transferred to PVDF membranes. Then, the membranes were blocked with 5% non-fat milk for 1 h at room temperature, probed with primary antibodies at 4 °C overnight, and incubated with secondary antibody for 2 h at room temperature. The intensities of the immunoreactive bands were quantified using SuperSignal chemiluminescent reagents (Pierce, Rockford, IL, USA).

### Tumor xenograft

The 6-week-old female BALB/c nude mice were used for xenograft transplantation model establishment. TFK-1 cells were injected subcutaneously (2 × 10^6^ cells/mouse) into the left axilla of mice. The mice were randomly allocated into two groups. When the tumor size reached 100 mm^3^(at about day 14), the mice were injected intraperitoneally with PG (5 mg/kg) or DMSO (6 mg/kg) in the PG or DMSO group twice a week. Tumour volume was monitored and documented every 4 days by the following formula: tumour volume = (Length × Width^2^)/2. The mice were sacrificed by intravenously injected pentobarbital sodium (150 mg/kg) at 28 days after PG or DMSO treatment. The tumor tissue was analysed.

### Immunohistochemistry (IHC)

The IHC examination was performed on the tumors resected from our xenograft transplantation mice. The tissue samples were fixed with formalin, embedded in paraffin, and cut into 5-µm-thick sections for further Hematoxylin-eosin(H&E) staining or IHC staining. The DAKO Autostainer system (Dako, Glostrup, Denmark) was used for IHC staining. The sections were observed using an Olympus microscope (Tokyo, Japan), and the images were analyzed using Image-Pro Plus 6.0.

### Statistical analysis

SPSS22.0 software (SPSS, Chicago, USA) was used for statistical analysis. Each experiment was conducted at least three times unless otherwise indicated. All data were presented as means ± standard error of the mean (SEM). Student’s t-test was performed to determine statistical differences. *P* < 0.05 was considered significant.

## Results

### PG promotes apoptosis by inhibiting the cellular activity of HuCCT-1 and TFK-1 cells

**Fig. 1:**
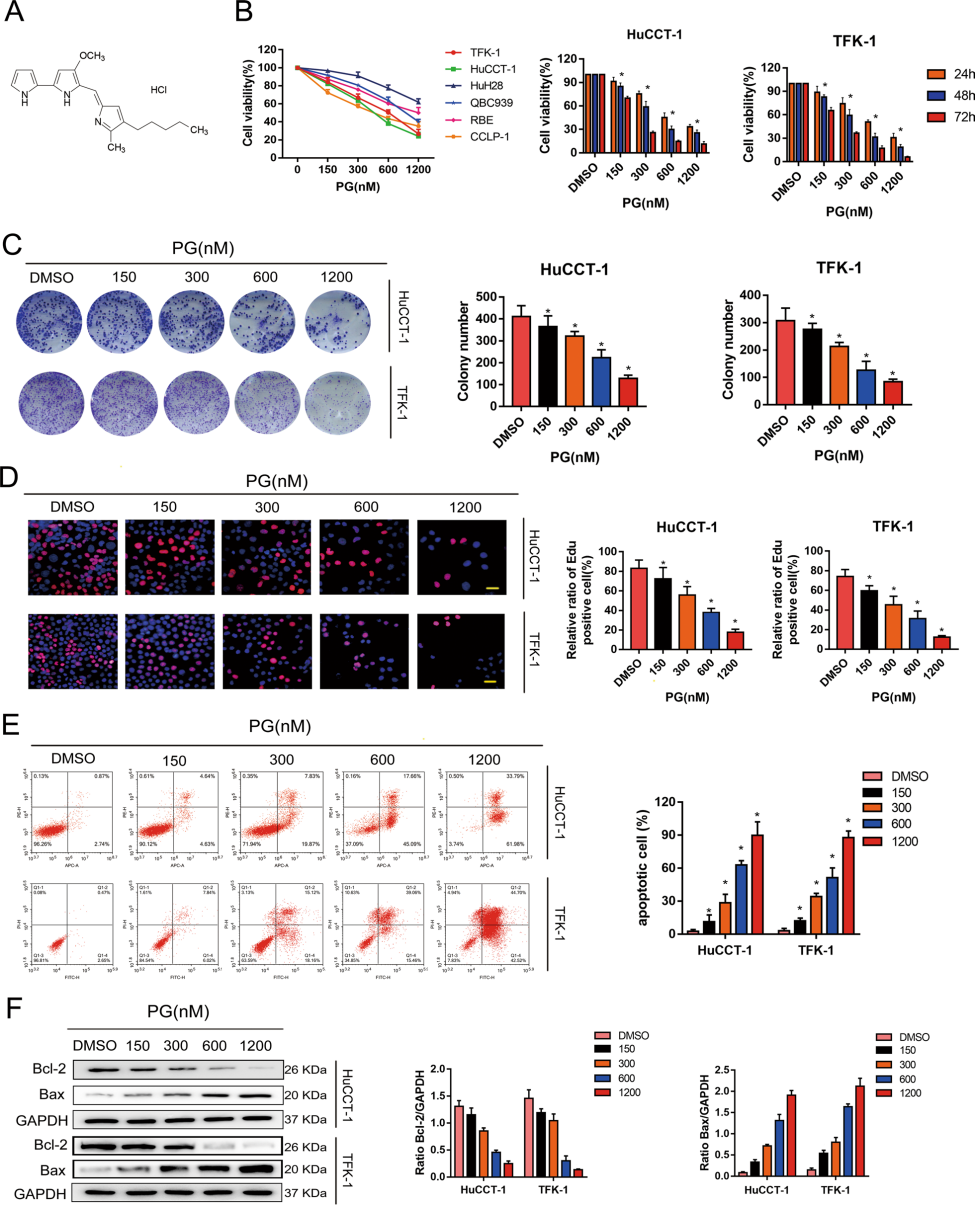
PG promotes apoptosis by inhibiting the cellular activity of HuCCT-1 and TFK-1. **A** Structure of PG. **B** HuCCT-1, TFK-1, HUH28, QBC939, RBE, and CCLP-1 cells were incubated with different concentrations of PG (150–1200 nM) for 24 h, followed by CCK-8 to determine cell viability. 0 nM PG is the DMSO vehicle. **C** Different concentrations of PG inhibited the colony-forming ability of HuCCT-1 and TFK-1 cells. **D** EdU analysis showed that PG inhibited the proliferation of HuCCT-1 and TFK-1 cells with increasing concentration compared to the DMSO control group. **E** Apoptosis of HuCCT-1 and TFK-1 cells by flow cytometry; **F** Expression levels of Bcl-2 and Bax were analyzed by western blot assay; *P<0.05

To evaluate whether PG inhibits cell proliferation in the CCA cell lines, CCK-8 assay was employed to detect the cell survival rate. The structure of PG was shown in Fig. 1A. Figure 1B shows increased inhibitory effects of PG on CCA cell proliferation in a concentration- and time-dependent manner. Next, we performed colony formation assay to explore whether PG inhibits the colony formation of TFK-1 and HuCCT cells (Fig. 1C). At the concentration of 600 nM and 1200 nM, the proliferation of both TFK-1 and HuCCT-1 cells was significantly inhibited by PG. Furthermore, we used EdU assay to investigate the proliferation capacity of TFK-1 and HuCCT-1 cells. The results displayed that there is a negative correlation between PG concentration and proliferation rate of CCA cells (Fig. 1D). Meanwhile, we found that the apoptotic effects of CCA cells was induced in a dose-dependent manner by PG (Fig. 1E). These findings were further supported by our following western blot results. Compared with the DMSO group, we found significantly downregulated expression in Bcl-2 but upregulated in Bax (Fig. 1F). In summary, PG inhibits cellular activity and promotes apoptosis in the HuCCT-1 and TFK-1 cells.

### PG inhibits autophagy via disturbing fusion of autophagosomes and lysosomes in CCA cells

**Fig. 2:**
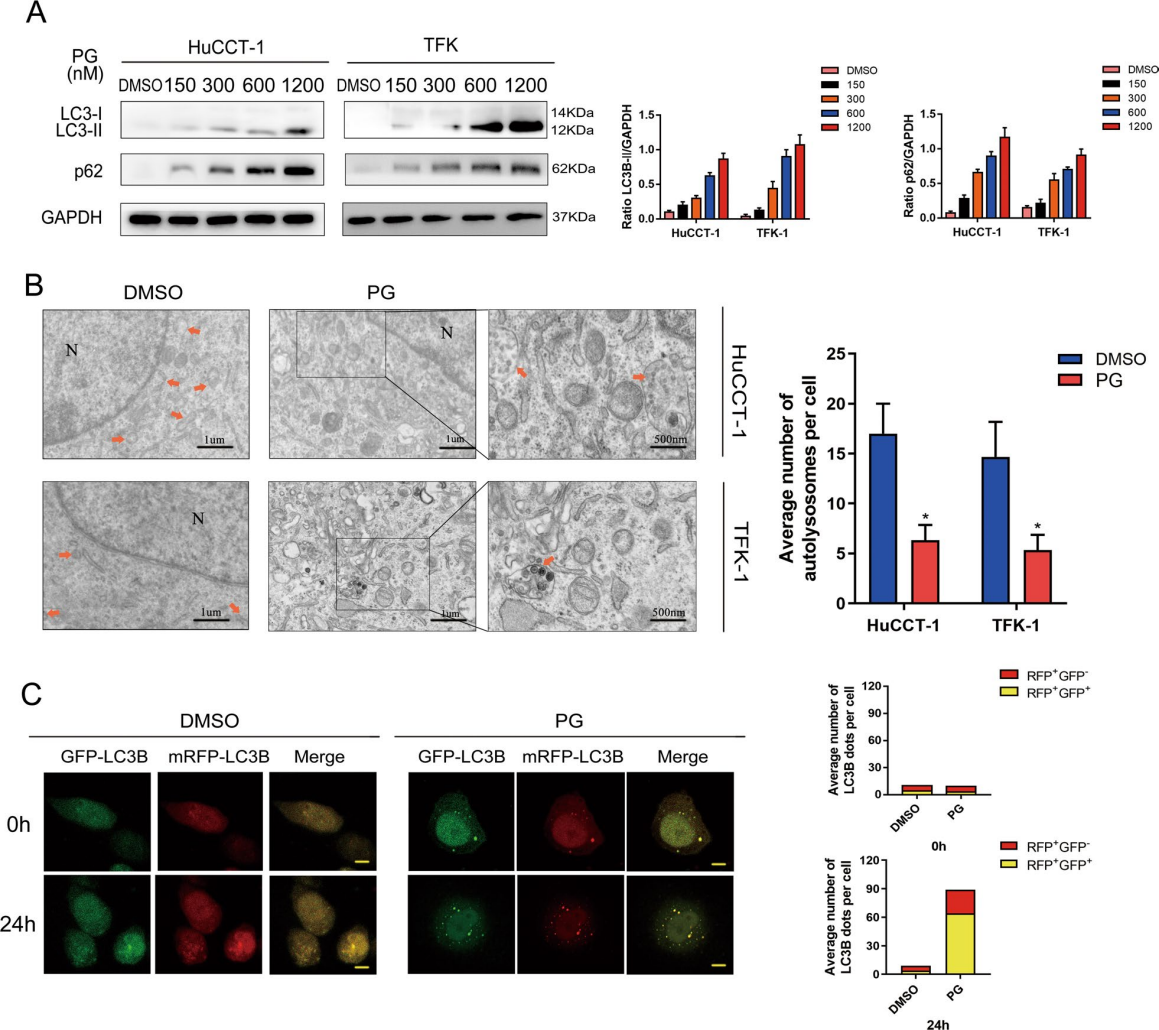
PG inhibits autophagy in CCA cells. **A** HuCCT-1 and TFK-1 cells were treated with different concentrations of PG for 24 h, and the expression levels of LC3B-II/I and P62 were compared by western blot analysis. **B** TEM was used to analyze the number of autolysosomes in HuCCT-1 and TFK-1 cells treated with DMSO (< 0.1%) or PG (600 nM) for 24 h. A magnified view of the electron photomicrograph shows characteristic autolysosomes. Arrowhead, autolysosomes; N, nuclear. **C** TFK-1 cells were transfected with GFP-mRFP-LC3B for 24 h and then incubated with DMSO (< 0.1%) or PG (600 nM) for 24 h. Autophagy flux was observed by confocal microscopy. Scale bar: 20 mm. All data are representative of three independent experiments; *P < 0.05

To explore whether PG is involved in the regulation of autophagy in CCA cells, we performed western blot assay. The result indicated a positive correlation between the expression level of both LC3B and p62 in HuCCT-1 and TFK-1 cells and the concentration of PG (Fig. 2A). Autolysosomes have only one limiting membrane which contain electron-dense cytoplasmic material or organelles at various stages of degradation. When the fusion of autophagosomes to lysosomes is blocked, the number of autolysosomes will decrease. Compared with the DMSO group, we observed less autolysosomes formed in the PG group under electron microscope (Fig. 2B). To measure autophagy flux, the HuCCT-1 and TFK-1 cells were transfected with a tandem mRFP-GFP-LC3 adenovirus. After PG treatment for 24 h, we observed accumulated green GFP puncta in TFK-1 cells, the co-localization of which with RFP (RFP+/GFP–) puncta became yellow autophagic puncta (RFP+/GFP+) (Fig. 2C). This phenomenon indicated that PG inhibits autophagy, particularly suppression fusion of autophagosomes and lysosomes, which is consistent with the results of the Western blot assay. All above results showed inhibitory effects of PG on the late stage of autophagy in CCA cells. The putative mechanisms may be related to biological processes regulating the combination of autophagy to lysosome.

### Inhibitory effect of PG on autophagy is mediated by SNAREs complex

**Fig. 3:**
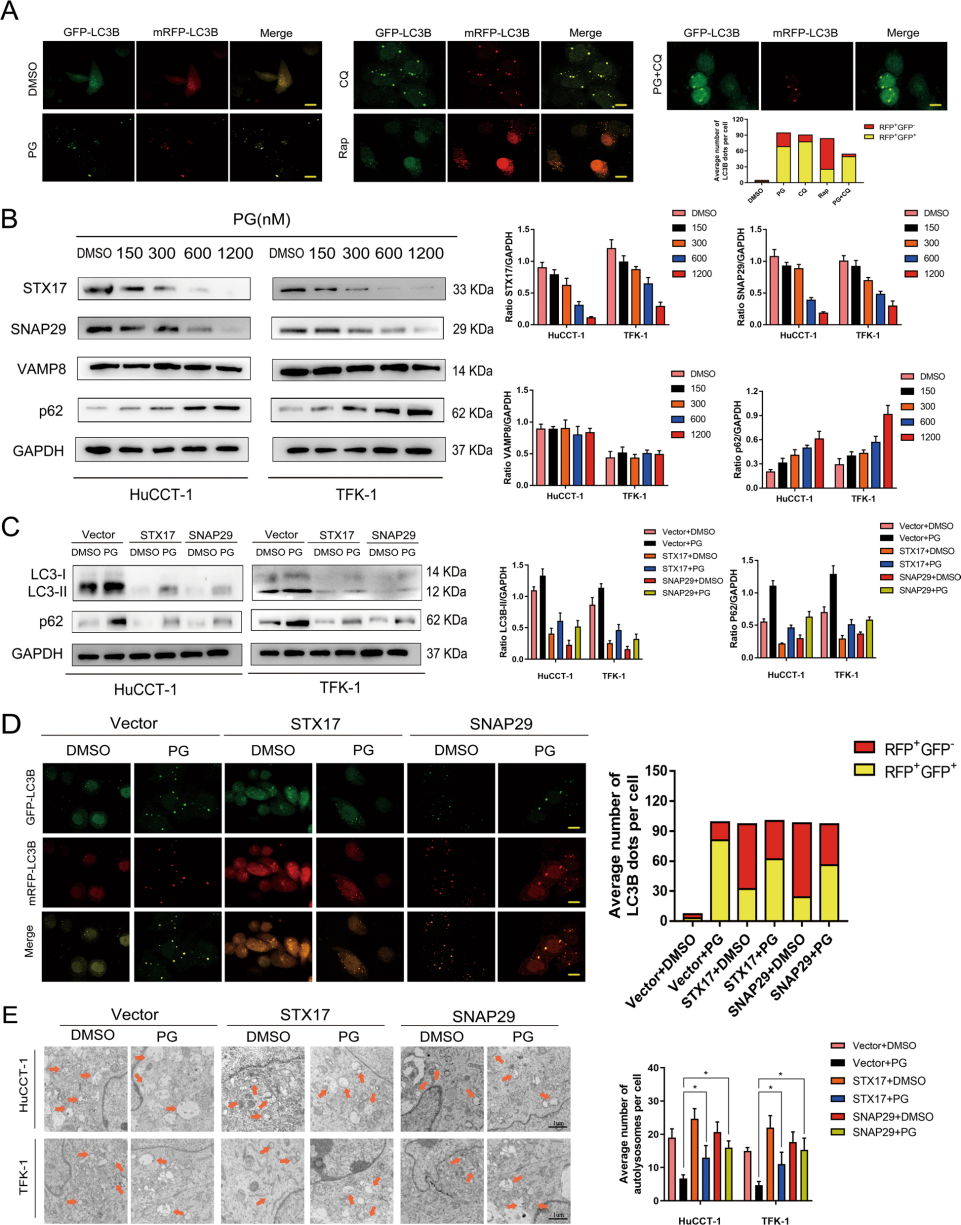
Inhibitory effect of PG on autophagy is mediated by SNAREs complex. **A** TFK-1 cells transfected with mRFP-GFP-LC3 were treated with DMSO (0.1%), PG (600 nM), CQ (10 µM), Rap (600 nM), and PG (600 nM) + CQ (10 µM), respectively, and the changes in green and red fluorescence were observed. Scale bar: 10 mm. **B** Western blot was used to detect the expression levels of SNARE proteins (STX17, SNAP29, and VAMP8) after treating CCA cells for 24 h with different concentrations of PG. **C** SNAP29 and STX17 were overexpressed in CCA cells, respectively. After the addition of PG or DMSO, the expression levels of LC3B-II/I and P62 autophagy proteins were detected by western blot analysis. **D** After transfecting TFK-1 cells with mRFP-GFP-LC3 for 24 h, autophagy flux was observed using a confocal microscope. **E** STX17 and SNAP29 were overexpressed in HuCCT-1 and TFK-1. After PG or DMSO treatment for 24 h, the changes in autolysosomes in CCA cells were observed by TEM. N, nuclear; arrows, autolysosomes; All data are representative of three independent experiments. *P < 0.05

To explore the underlying mechanism about inhibitory effect of PG on autophagy, we treated CCA cells with Rapamycin (RAP) and Chloroquine (CQ), respectively. We observed increased RFP in RAP-treated CCA cells. On the other hand, in CCA cells treated with either CQ or PG, GFP was elevated. When CCA cells treated with CQ + PG, the proportion of GFP has increased further (Fig. 3A). We used western blot assay to evaluate the effect of PG on the expression of intracellular SNAREs complex. As is shown in Fig. 3B, the expression of VAMP8 was not affected by the increased concentration of PG. However, the expression of STX17 and SNAP29 was altered significantly, indicating that the autophagy regulatory axis is comprised of PG, STX17, and SNAP29. Then, we used western blot assay to evaluate the expression level of P62 and LC3B-II in STX17- or SNAP29-overexpressed CCA cells. Compared to the PG-treated group, we found significantly decreased expression of both P62 and LC3B-II in STX17- or SNAP29-overexpressed group, indicating an inhibitory effect of PG on autophagy (Fig. 3C). After transfection with mRFP-GFP-LC3 adenovirus and PG treatment, we measured the autophagy flux in the vector group, STX17-overexpressed group, and SNAP29-overexpressed group. RFP was significantly increased in both STX17- or SNAP29-overexpressed groups compared to the vector group (Fig. 3D). The number of autolysosomes in each group was then measured using TEM, a significantly increased number of autolysosomes was observed in both STX17- and SNAP29-overexpressed groups compared to the vector group (Fig. 3E). These results suggest that PG blocked the late stage of autophagy in CCA cells through the SNAREs complex pathway.

### Inhibitory effect of PG on CCA cell activity is mediated by SNAREs complex

**Fig. 4:**
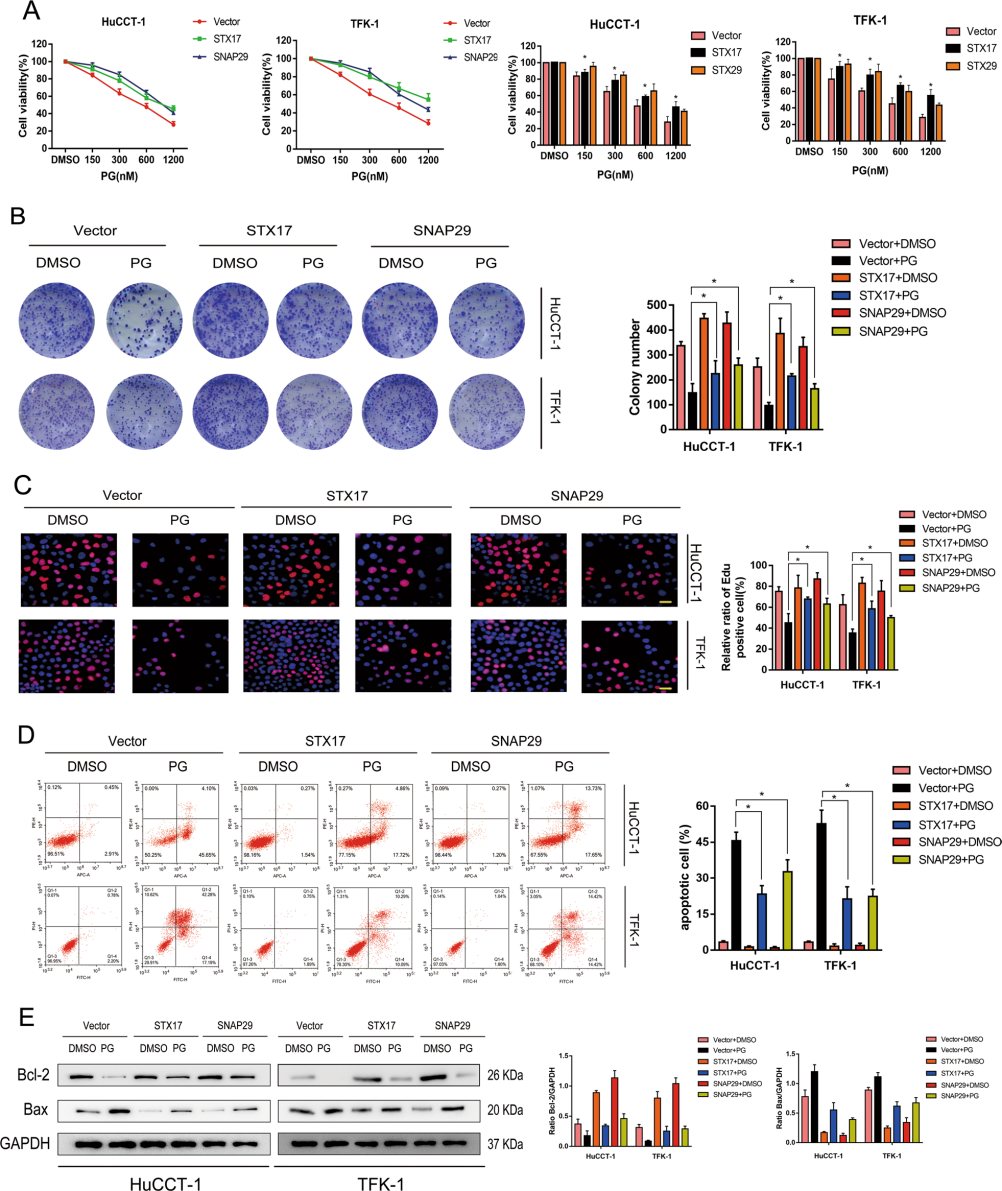
Inhibitory effect of PG on CCA cell activity is mediated by SNAREs complex. **A**–**E** HuCCT-1 and TFK-1 cells were infected with overexpressed STX17 and SNAP29 adenovirus, respectively. Cells were evaluated as follows: **A** Cell viability with CCK-8 after the addition of DMSO or PG. **B** Clone formation test to determine the rate of clone formation. **C** EDU test to detect cell proliferation. **D** Apoptosis of HuCCT-1 and TFK-1 cells by flow cytometry. **E** Western blot assay was used to detect the expression of apoptosis-related proteins; *P < 0.05

To explore whether the inhibitory effect of PG on the proliferation of CCA cells is mediated by SNAREs complex, we performed CCK-8, colony formation, and EDU assays to assess cell activity in PG-treated vector, PG-treated STX17 overexpressed, and PG-treated SNAP29-overexpressed groups. Under treatment with PG, the cell proliferation capacity was decreased, while it was significantly enhanced after STX17- or SNAP29-overexpressed (Fig. 4A–C). Then, we performed flow cytometric analysis in each group to assess the CCA cell apoptosis rate and found that the acceleration effect of PG on CCA cell apoptosis could be inhibited by the overexpression of STX17 or SNAP29 (Fig. 4D). Furthermore, the result of western blot showed that compared to the vector group, Bcl-2 was upregulated but Bax was downregulated in STX17- or SNAP29-overexpressed groups (Fig. 4E). This phenomenon supported that PG inhibits the activity of CCA cells by interacting with SNAREs complex.

### Anti-tumor activity of PG in xenograft animal model

**Fig. 5:**
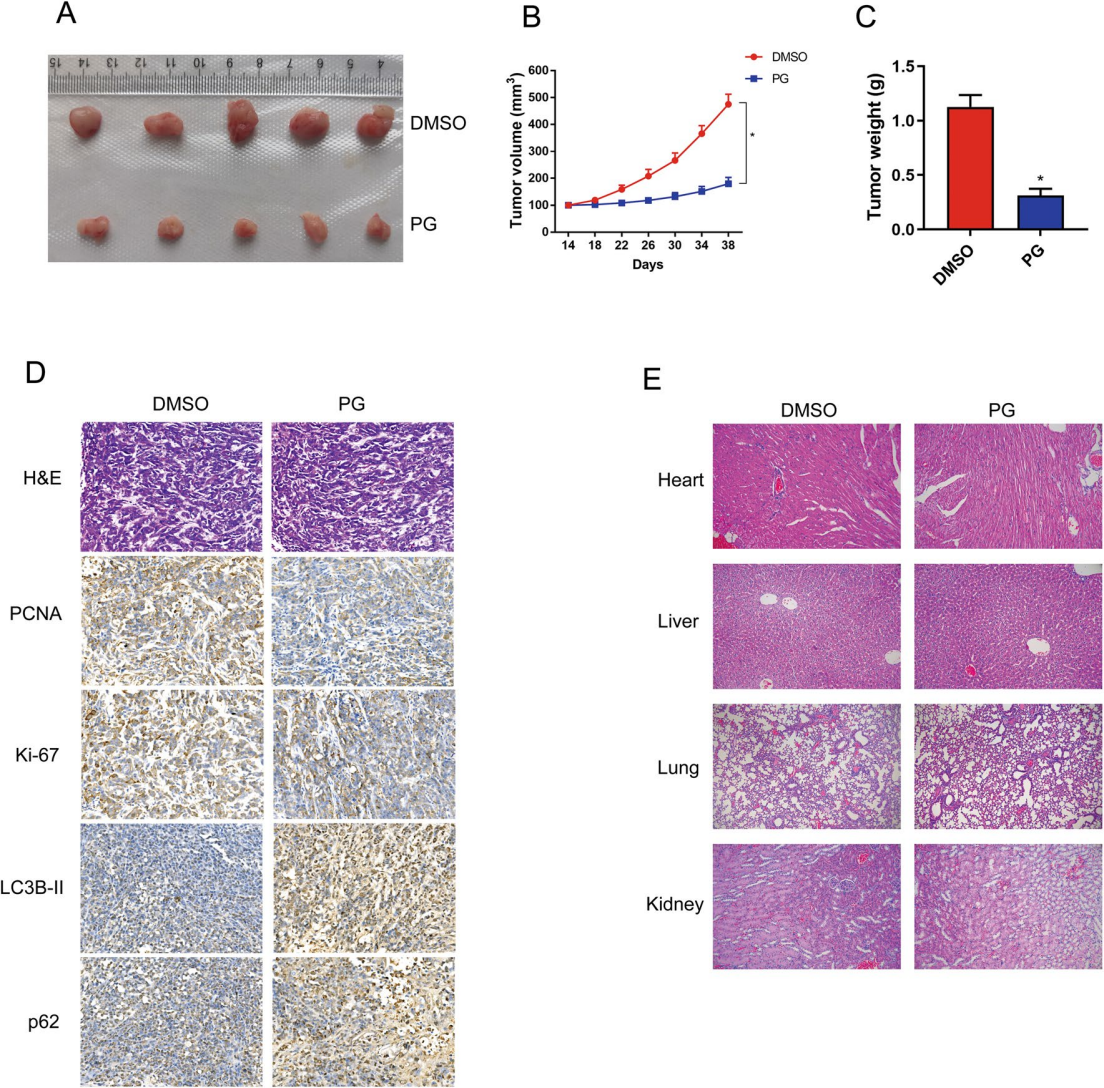
Anti-tumor activity of PG in xenograft animal model. **A** BALB/c nude mice were inoculated subcutaneously with TFK-1 cells. When the tumor reached 100mm^3^, the nude mice (n = 5) were treated with DMSO or PG for 24 days. Representative images of tumor-bearing mice, **B**, **C** tumor weight and volume at different time points. **D** IHC staining was used to detect PCNA, Ki-67, LC3-II, and p62 proteins in tumor tissues of DMSO and PG groups. **E** H&E staining of vital organs in nude mice. Scale bar: 50 mm; *P < 0.05

Finally, we explored whether anticancer activity in vivo was inhibited by PG using a xenograft tumour model. The xenograft mice were respectively treated with DMSO (6 mg/kg) and PG (5 mg/kg) for 24 days. As shown in Fig. 5A–C, PG-treated tumors grew more slowly than tumors in the DMSO group. The IHC analysis revealed that compared with the DMSO group,the expression level of PCNA and Ki-67 was downregulated in the PG-treated group. However, a higher expression of LC3B-II and P62 was found in the PG-treated group than in the DMSO group (Fig. 5D). In addition, organs H&E staining results revealed that there is no distinct difference between PG-treated group and DMSO-treated group (Fig. 5E). These results demonstrate that PG shows anti-tumor activity in vivo through regulating autophagy.

## Discussion

Autophagy is a process of self-digestion in eukaryotic cells, which degrades damaged organelles and proteins to maintain normal cell activities [18, 24]. After tumor cells undergo autophagy, they provide amino acids, fatty acids, and energy, which helps the tumor cells survive under severe conditions, such as ischemia and hypoxia [25, 26]. Although autophagy is essential for tumor development, the corresponding targeted autophagy therapy has not been clearly studied. Recent studies about the molecular mechanism of autophagy are mainly focused on the initiation, extension and fusion of autophagosomes. Perera et al. suggested that Ras-driven pancreatic cancers activate transcription programs for autophagy and lysosomal biogenesis by promoting the nuclear localization of the master regulatory microphthalmia (MiT/TFE)family [27]. Ferguson et al. reported that the autophagy-lysosome system has an important effect on promoting tumor growth [28]. Yu et al. found that the regulation of autophagy process is quite vital, especially the fusion process of autophagosome and lysosome [29]. Therefore, based on the current research results, numerous autophagy pathways can be used as targets for exploring their potentiality as clinical treatment options. Xu et al. demonstrated that inhibition of late-stage autophagy resulted in the death of pancreatic cancer cells [30]. Gao et al. reported in their study that by blocking the Akt/mTOR pathway, they successfully induced the fusion of autophagosome and lysosome, which promotes autophagy and finally leads to the death of ovarian cancer cells [31]. According to the studies mentioned above, the effects of anti-tumor agents on autophagy and its molecular mechanism is unclear, hence, further investigation is needed.

Our present study showed that PG regulated the activity of CCA cells through autophagy. PG is a secondary metabolite produced by many bacteria with serious biological functions. Zhao et al. demonstrated that PG inhibits autophagy and reduces the viability of colorectal cancer cells [32]. Ji et al. showed that PG inhibits autophagy and induces apoptosis in K562 cells via the ERK signaling pathway [33]. The current data showed that the activity and colony formation rate of CCA cells decreased significantly after the addition of PG at various concentrations, and the apoptosis rate showed an upward trend. Western blot analysis showed an increased expression of apoptotic protein Bax and a decreased expression of Bcl-2. Thus, we concluded that PG mediated the activity of CCA cells through autophagy, which is verified by proliferation and apoptosis assays.

The process of autophagy is comprised of initiation, nucleation, extension, maturation, and degradation [34]. Furthermore, we identified which process could PG impact on autophagy. According to the study by Katsuragi et al., when autophagy is inhibited, the expression of p62 is increased while the expression of LC3B-II showed a downward trend [35]. Interestingly, we found that with increasing concentration of PG, the expressions of LC3B-II and p62 were increased. Meanwhile, PG were found to inhibit the maturation of autophagy and result in more autophagosomes (yellow) in CCA cells in a manner similar to CQ. We consider that PG may block the fusion of autophagosome and lysosome. Several studies have reported that the fusion of autophagosomes and lysosomes is correlated with SNAREs complex, which is composed of STX17, SNAP29, and VAMP8 [36]. STX17 is located in the outer layer of the autophagosome membrane, where it recruits SANP29 and binds to VAMP8 in the lysosome and promotes the binding of autolysosomes [37]. Li et al. inhibited autophagy by regulating SNAREs complex in pancreatic cancer cells [38]. We proved that PG regulated the protein expression level of STX17 and SNAP29 by western blot. Autophagy flux analysis reveals PG disturbing fusion of fusion of autophagosomes and lysosomes.

**Fig. 6:**
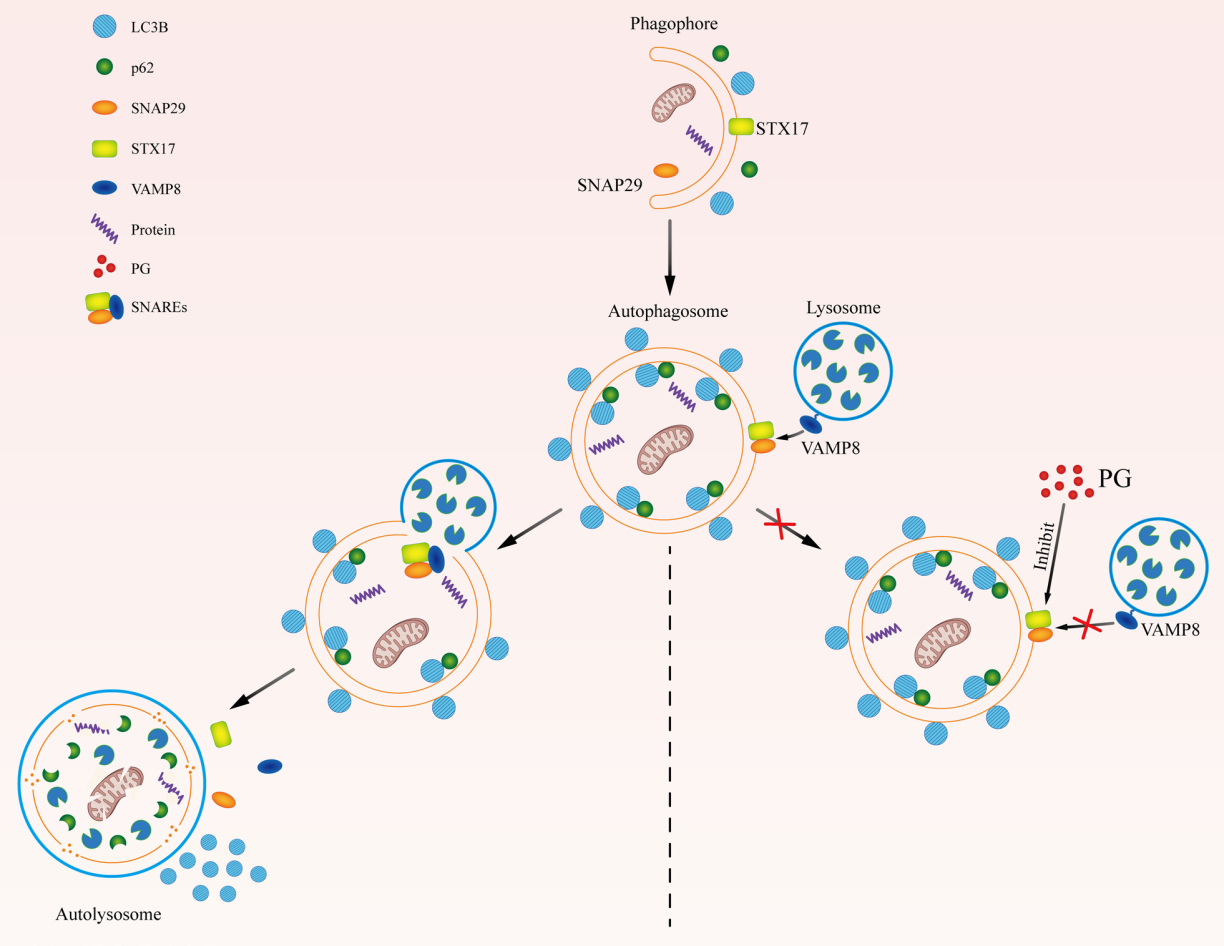
Schematic diagram of the effect of PG on inhibiting autophagy in CCA cells. During autophagy, LC3B and P62 in autophagosomes will be degraded by hydrolase in lysosomes along with the fusion of autophagosomes with lysosomes. STX17, SNAP29 and VAMP8 constitute complex of SNAREs, which plays a key role in this fusion process. When the expression of STX17 and SNAP29 was inhibited by PG, STX17 and SNAP29 could not effectively form SNARE complex with VAMP8. And the fusion of autophagosome with lysosome was then inhibited, which subsequently result in failure of degradation of LC3B and P62

In summary, PG inhibits STX17 and SNAP29 in the SNAREs complex (Fig. 6) and subsequently inhibits the generation of autolysosome. Meanwhile, PG reduced autophagy activity by inhibiting late stage of autophagy and exerted anti-tumor activity in CCA. Therefore, our study may provide a potential promising treatment agent for the treatment of advanced CCA.

## Data Availability

All data generated or analyzed during this study are included in this published article.
